# Usability and Usefulness of a Symptom Management Coaching System for Patients With Cancer Treated With Immune Checkpoint Inhibitors: Comparative Mixed Methods Study

**DOI:** 10.2196/57659

**Published:** 2025-01-23

**Authors:** Savannah Lucia Caterina Glaser, Itske Fraterman, Noah van Brummelen, Valentina Tibollo, Laura Maria Del Campo, Henk Mallo, Sofie Wilgenhof, Szymon Wilk, Vitali Gisko, Vadzim Khadakou, Ronald Cornet, Manuel Ottaviano, Stephanie Medlock

**Affiliations:** 1 Department of Medical Informatics Amsterdam UMC - University of Amsterdam Amsterdam Netherlands; 2 Methodology & Digital Health Amsterdam Public Health Amsterdam Netherlands; 3 Department of Psychosocial Research and Epidemiology The Netherlands Cancer Institute Amsterdam Netherlands; 4 Digital Health Amsterdam Public Health Amsterdam Netherlands; 5 Laboratory of Informatics and Systems Engineering for Clinical Research Istituti Clinici Scientifici Maugeri SpA SB IRCCS Pavia Italy; 6 Associazione Italiana Malati di Cancro Rome Italy; 7 Department of Medical Oncology Antoni van Leeuwenhoek Amsterdam Netherlands; 8 Institute of Computing Science Poznan University of Technology Poznan Poland; 9 BITSENS JSC Vilnius Lithuania; 10 Life Supporting Technologies Universidad Politécnica de Madrid Madrid Spain; 11 Methodology & Aging & Later Life Amsterdam Public Health Amsterdam Netherlands

**Keywords:** oncology, usability, usefulness, symptom management, coaching system, patients with cancer, immune checkpoint inhibitors, comparative qualitative study, medication, eHealth applications, caregivers, cancer treatment, patient education, well-being interventions, acceptability, melanoma, renal cell carcinoma, immunotherapy

## Abstract

**Background:**

The prognosis for patients with several types of cancer has substantially improved following the introduction of immune checkpoint inhibitors, a novel type of immunotherapy. However, patients may experience symptoms both from the cancer itself and from the medication. A prototype of the eHealth tool Cancer Patients Better Life Experience (CAPABLE) was developed to facilitate symptom management, aimed at patients with melanoma and renal cell carcinoma treated with immunotherapy. Better usability of such eHealth tools can lead to improved user well-being and reduced risk of harm. It is unknown for usability evaluations whether certain usability problems would only be evident to patients whose condition closely resembles the target population, or if a broader group of patients would lead to the identification of a broader range of potential usability issues.

**Objective:**

This study aims to evaluate the CAPABLE prototype by conducting tests to assess usability, user experience, and perceived acceptability among end users, and to assess any agreements or differences in the results of our wide range of participants.

**Methods:**

This usability study was executed by interviewing participants with a melanoma or renal cell carcinoma diagnosis who have received immunotherapy and participants without direct experience with the targeted cancer types who have not received immunotherapy. Participants were asked to review the concept of the tool, perform think-aloud tasks, and complete the System Usability Scale and a Perceived Usefulness questionnaire. Usability problems were extracted from the interview data by independent coding and mapped to an eHealth Usability Problem Framework.

**Results:**

We included 21 participants in the study, aged 29 to 73 years; 13 participants who had received immunotherapy and 8 participants who had not received immunotherapy. In total, 76 usability problems were identified. A total of 22 usability problems were in the task-technology fit category of the usability framework, mostly regarding the coaching and symptom functionality of the prototype. Critical problems regarding the symptom monitoring functionality were mainly found by participants who had received immunotherapy. For 8 out of 10 statements in the Perceived Usefulness questionnaire, more than 75% of participants agreed or strongly agreed. The overall mean System Usability Scale score was 80 out of 100 (SD 11.3).

**Conclusions:**

Despite identified usability issues, participants responded positively to the Perceived Usefulness questionnaire regarding the evaluated tool. Further analysis of the usability problems indicates that it was essential to include participants who matched the target end users. Participants treated with immunotherapy, specifically with previous experience in immune-related adverse events, encountered critical problems with symptom reporting that would not have been identified if these participants were not included. For other tasks and functionalities, it seems likely that loosening the inclusion criteria would have resulted in sufficient feedback without critical missing usability issues.

## Introduction

The prognosis for patients with several types of cancer has substantially improved following the introduction of immune checkpoint inhibitors, a novel form of immunotherapy that incites the patient’s own immune system to attack the cancer cells [1-4]. The treatment has improved prognosis, but also often incites side effects, ranging from mild to potentially life-threatening. In addition, patients may experience symptoms from the cancer itself, as well as the stress of the disease and treatment on their mental well-being. Consequently, patients may experience a diminished health-related quality of life [2-5]. Previous research has shown that patients with cancer often have unmet care and information needs during their treatment and follow-up. eHealth apps may support health providers in addressing these needs [6,7].

For example, eHealth apps in cancer treatment are used to facilitate timely symptom reporting by the collection of patient-reported outcomes, by providing information for patients and caregivers on diagnosis, treatment, and side effects, and by giving patients access to home interventions for physical and mental well-being [8-12]. While there is conclusive evidence on the impact of eHealth on perceived support and knowledge levels, there are inconsistent findings for outcomes related to quality of life, self-management, and physical or mental well-being [6,13,14]. These potential benefits of eHealth apps are partially dependent on their ease of use, Perceived Usefulness, and eventual user acceptance [15,16].

It is known that user-centered design (UCD) processes for these types of eHealth apps may benefit system usability and user acceptance [16-18]. Better usability can lead to benefits such as enhanced user well-being and reduced risk of harm [19]. A significant part of UCD is evaluating apps using a usability evaluation method (UEM). Most UEMs highlight the importance of doing so with the intended end users of the product in order to accurately extract and understand the usability problems of target users [20]. The International Organization for Standardization standard for health and wellness apps states if the app is specifically designed to cater to individuals with a particular health condition, testing should involve participants with that condition [21]. However, the characteristics of the intended end users for health apps might not be strictly defined, or the intended end users might consist of a broadly defined, heterogeneous patient population [22]. Thus, it is not always clear which user characteristics should be considered when recruiting representative participants. In addition, it may be difficult for researchers to find patients who fit a particular profile, forcing them to adjust their inclusion criteria.

Prior to this study, we developed a prototype of the eHealth tool Cancer Patients Better Life Experience (CAPABLE). This tool facilitates symptom reporting, patient education, and well-being interventions [23]. The prototype was developed in an iterative manner based on UCD principles [6]. At the start of prototype development, preliminary and semistructured interviews were conducted with patients, caregivers, and health care professionals, to elicit their specific support needs and requirements for an eHealth tool [7]. These requirements were translated into functionalities of the prototype, followed by a preliminary usability test using heuristic evaluation.

The CAPABLE tool is intended for melanoma and renal cell carcinoma patients treated with immunotherapy, and their health care providers. During development, we aimed to consider various aspects of implementation for these different patient populations in different countries. The pilot study of CAPABLE will focus on these specific patient populations [24]. However, it is intended to be useful for a more general population of patients with cancer who are undergoing treatment. It is not known whether certain usability problems would only be evident to patients whose condition closely resembles patients in the trial, or if a broader group of patients would lead to the identification of a broader range of potential usability issues. This is particularly relevant for systems like CAPABLE, intended for use by patients with serious health complaints, who ethically should only be asked to participate in tasks where their time and effort are truly needed.

Therefore, we aimed to evaluate the CAPABLE prototype by conducting tests to assess usability, user experience, and perceived acceptability among end users. A secondary objective of the study is to assess any agreements or differences in the results of our wide range of participants in the usability studies, considering the target population of our eHealth intervention (patients with melanoma and renal cell carcinoma treated with immunotherapy) and participants that are part of a broader population (patients with cancer with other types of cancer and informal caregivers).

## Methods

### Overview

The CAPABLE prototype was developed by a multidisciplinary Consortium, as part of the CAPABLE Project [23]. The overall aim of the project is to explore the effect, usability, and feasibility of the CAPABLE tool in a pilot study with patients with melanoma and renal cell carcinoma, during treatment with immune-checkpoint inhibitors [24]. This usability study was performed as part of the UCD process, through a think-aloud approach where participants were asked to execute scenario-based tasks using the CAPABLE prototype [20,25]. We followed the COREQ (Consolidated Criteria for Reporting Qualitative Research) checklist for reporting our results (Multimedia Appendix 1).

### The CAPABLE Prototype

**Figure 1:**
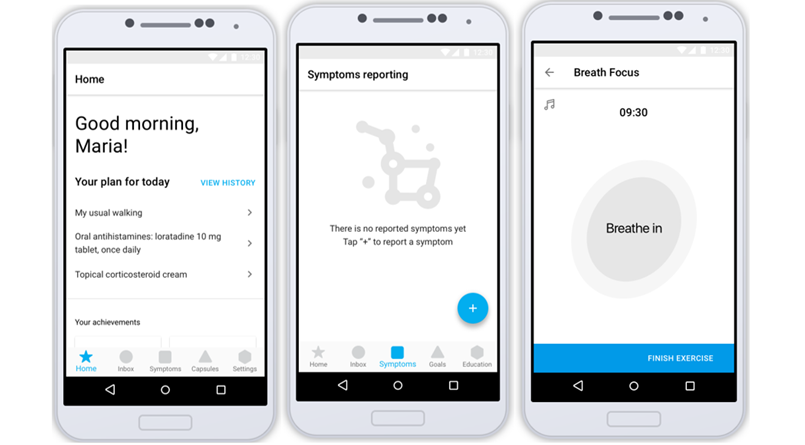
Excerpt of screens from the CAPABLE mobile app prototype evaluated during the usability study. CAPABLE: Cancer Patients Better Life Experience.

The CAPABLE prototype consists of a mobile app for patients and a web-based dashboard for their health care providers. This usability study focuses on the mobile app for patients. Software from Invision was used to create a full clickable prototype of the CAPABLE app. See Figure 1 for an excerpt of screens from the CAPABLE prototype app screens [26].

The mobile app for patients consists of five different sections to facilitate symptom reporting, well-being interventions, and patient education (Table 1).

**Table 1:** Sections of the CAPABLE mobile app with their goals and content for supporting patients treated with immunotherapy, including symptom reporting, well-being interventions, and patient education.

Sections	Goals and contents
Home page	Contains a daily plan for patients to follow, including their hospital appointments, links to patient questionnaires, and suggestions for well-being interventions.
Inbox	Contains messages, recommendations, and reminders regarding their symptoms and well-being interventions.
Symptom reporting	Allows users to report their symptoms and provides feedback based on implemented computer-interpretable clinical guidelines for the management of symptoms related to immunotherapy side effects. Users report symptoms by selecting from a predefined set of descriptions that detail both the severity of symptoms and their impact on daily activities. After reporting, they receive feedback such as self-care instructions or advice to contact their health care provider.
Goals	Provides well-being goals such as improving sleep, physical well-being, and mental well-being, and offers corresponding theory-driven digital behavior change interventions including breathing exercises, meditation, and walking activities [27].
Education	Contains information about melanoma and renal cell carcinoma, treatments including targeted therapy and immunotherapy, side effects, supportive care, and nutrition.

### Recruiting and Inclusion

Three recruiting organizations participated in the study, the Netherlands Cancer Institute-Antoni van Leeuwenhoek (NKI-AvL) in The Netherlands, Istituti Clinici Scientifici Maugeri (ICSM) in Italy, and the Italian Association of Cancer Patients, Relatives and Friends (AIMAC).

We aimed to recruit three participant groups; individuals diagnosed with melanoma previously treated with immunotherapy, those with renal cell carcinoma previously treated with immunotherapy, and participants without direct experience with the targeted cancer types nor with immunotherapy (thus patients with other types of cancer or informal caregivers). For each participant group, our goal was to recruit seven to nine participants. We did not specifically aim for data saturation as we considered the recommended sample sizes found in the literature, which range from five to ten participants. We chose seven to nine participants per group to reduce the risk of missing usability problems while being sensitive to time and resource constraints [28,29]. The inclusion criteria and recruitment strategies are described in Table 2.

**Table 2:** Inclusion criteria and recruitment strategies across the three participating centers.

	NKI-AvL^a^	ICSM^b^	AIMAC^c^
Participant	Adult patients	Adult patients	Adult AIMAC members or their informal caregivers
Diagnosis	High risk (resectable stage III) or advanced (stage IV and unresectable stage III) melanoma	Renal cell carcinoma	Any type of cancer
Treatment	During or after treatment with immune checkpoint inhibitors	During or after treatment with immune checkpoint inhibitors	Any type of treatment
Language	Sufficient understanding of the Dutch language	Sufficient understanding of the Italian language	Sufficient understanding of the Italian language
Recruitment	Purposive sampling strategy to obtain a sample that varied in age Participants were invited by their treating clinician, face-to-face or by telephone	Purposive sampling strategy to obtain a sample that varied in age Participants were invited by their treating clinician, face-to-face or by telephone	Open enrollment recruitment from volunteering patient network

^a^NKI-AvL: the Netherlands Cancer Institute-Antoni van Leeuwenhoek.

^b^ICSM: Istituti Clinici Scientifici Maugeri in Italy.

^c^AIMAC: the Italian Association of Cancer Patients, Relatives, and Friends.

### Ethical Considerations

This study was approved by the medical ethics committee of Amsterdam Universitair Medisch Centrum (Amsterdam; ID 2023.0944). Written informed consent was obtained from all participants prior to the interviews. All data collected for this study were pseudonymized with a new identifier. The research participants were not compensated for their study participation.

### Interviews

#### Overview

The interviews were performed by two research teams. The interviews at NKI-AvL were performed in Dutch by two female PhD candidates with prior interviewing experience (IF and SLCG). The interviews at ICSM and AIMAC were performed in Italian by a female researcher (VT), a male senior researcher (MO), and a male research assistant with previous experience as a health care professional (Federico Dagostin). Two participants from NKI-AvL had participated in a previous interview with the NKI-AvL research team to elicit specific needs and requirements for the CAPABLE tool. The researchers had no clinical relationship with nor did know the remaining participants. A collaborative training session was held with all interviewers to streamline the UEMs used in the interviews as much as possible.

Due to the COVID-19 pandemic, interviews were done digitally using videoconferencing tools Teams (Microsoft Corp) and Zoom (Zoom Video Communications). The interviews were recorded using the screen- and audio-capture functionalities of these tools. The planned duration of the interviews was 45-60 minutes. The interviews were conducted in three phases: (1) the introduction, where participants were asked their opinion on the CAPABLE concept; (2) completing think-aloud tasks, and (3) filling in questionnaires. Participants were aware that the interviewers were researchers involved in the development of the CAPABLE prototype. See Multimedia Appendix 2 for the interview protocol.

#### Phase I: Introduction

The interview started with a few questions regarding patient demographics, and their previous experience with smartphones and technology. This was followed by a short presentation about the CAPABLE system and a few open-ended questions regarding the participant’s opinion on the proposed goals and functionalities of the mobile app. Then, the interviewer explained the concept of the think-aloud phase of the interview. The participants were informed that the purpose of the think-aloud tasks was to evaluate the app’s performance and that the method required them to talk about what they were doing and thinking whilst using the app.

#### Phase II: Think-Aloud Tasks

The participants were asked to complete five tasks in total. These tasks were developed in cooperation with research team members and the developer. The tasks were (1) go through the introduction, check notifications, and report an activity; (2) report an itch symptom in the patient role; (3) report a fever symptom in the caregiver role; (4) find and perform a deep breathing exercise; and (5) find and review information about skin toxicity.

After performing the tasks, participants were asked about their final opinions, suggestions, or functionalities that they missed in the app.

#### Phase III: Questionnaires

Next, we administered the System Usability Scale (SUS) survey, a 10-item questionnaire aimed to assess the usability of a system considering effectiveness, efficiency, and satisfaction [30]. Finally, the participants were asked to fill out an 11-item questionnaire on Perceived Usefulness, based on the Technology Acceptance Model [31]. For both questionnaires, each item contains a statement that the participant is asked to rate based on a 5-point scale, ranging from strongly agree to strongly disagree (Multimedia Appendix 3).

### Data Analysis

#### Participant Characteristics

The participants’ characteristics were analyzed using descriptive statistics. We divided participants into two groups: treated with immunotherapy and not treated with immunotherapy.

#### Task Completion

We assessed the effectiveness of the participants performing the tasks according to three measures: (1) completed with ease, (2) completed with difficulty, and (3) failed to complete. We defined completed with difficulty as completed while needing to receive hints from the interviewer. Giving hints was done to ensure that the participant would have the opportunity to review all the content of the app and allow the researchers to obtain additional information about the usability of the app regardless of the completion status of the task.

#### Usability Problems

The Dutch interview recordings at NKI-AvL were transcribed verbatim (SLCG) as source data for our coding and analysis of the usability problems. During the Italian interviews at ICSM and AIMAC, notes were made by the interviewer that summarized the participants’ feedback. These notes were revised and completed after the interview upon reviewing the recording. The transcripts and notes were not returned to the participants.

As two research teams performed the interviews and the data used to code the interviews varied (ie, transcripts and summarizing notes), we conducted a data quality check. Two Italian interviews and two Dutch interviews were transcribed and independently coded by researchers from both research teams (SLCG and VT). The codes based on the transcript were compared to those based on the summarizing notes of the Italian interviews to evaluate their consistency. In addition, the independently coded interviews were compared to ensure whether the interpretation of the data was similar between the two research teams. Transcripts and notes were considered comparable if both reviewers agreed that there were no substantial differences or omissions in the code’s results.

#### Coding and Analysis of Interview Recordings

**Figure 2:**
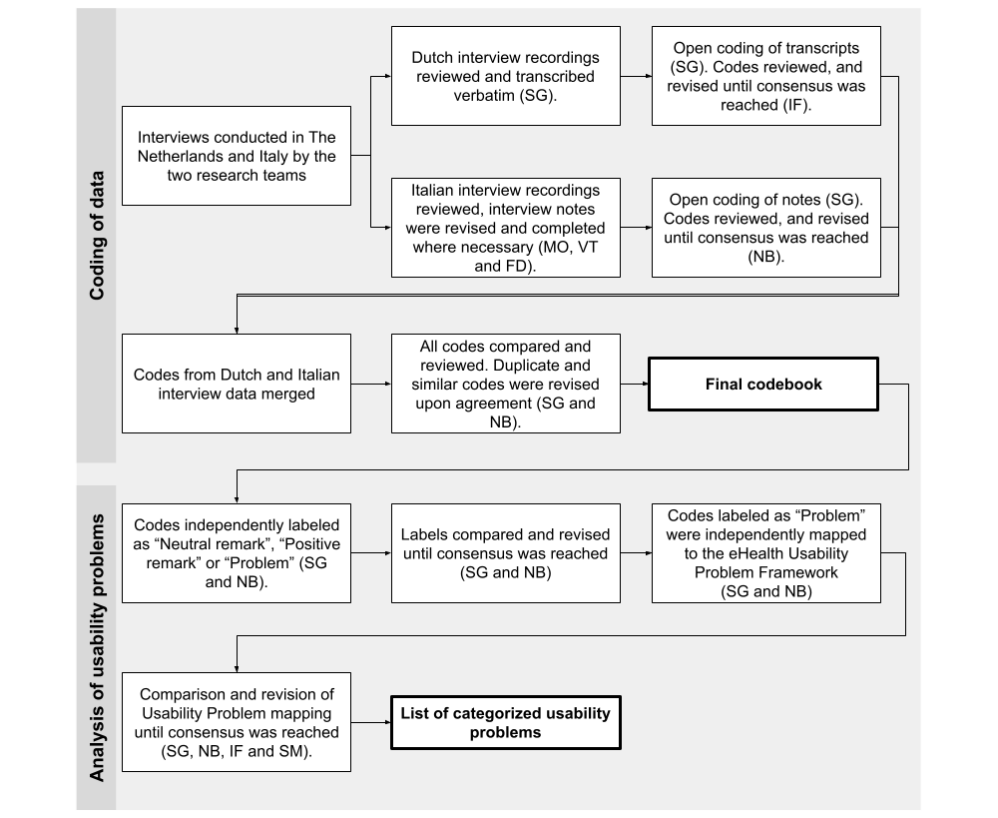
Steps executed for open coding of the usability interview transcripts and notes and analysis of the usability problems by mapping to the eHealth Usability Problem Framework. FD: Federico Dagostin.

Several steps were taken to code and analyze the interview recordings. This was executed by the same researchers who conducted the Dutch interviews (SLCG and IF), plus NB, an MSc in Medical Informatics. The coding and analysis were done in English. See Figure 2 for an overview of these steps.

First, the interviews were open-coded by SLCG. The codes from the Dutch and Italian interviews were reviewed by IF and NB, respectively, until a consensus was reached. After merging the data and codes from the Dutch and Italian interviews, an extra revision step was executed to ensure that duplicate and similar codes were revised, upon agreement of SLCG and NB. This resulted in the final codebook. All codes in the codebook were independently labeled by SLCG and NB as “Positive remark,” “Neutral remark,” or “Problem” to allow us to focus on the usability problems indicated by the participants. Then, we mapped the “Problem” codes in the codebook to an eHealth Usability Problem Framework developed by Broekhuis et al [32]. This framework specifies 21 usability factors in the following categories: basic system performance, task-technology fit, accessibility, interface design, navigation and structure, information and technology, guidance and support, and satisfaction. This mapping process was done by SLCG and NB, in three different cycles, independently mapping and comparing the mappings, reaching consensus. Any discrepancies were discussed with IF and SM.

To gain insight into the usability problems and the frequency with which they were encountered, we counted the distinct problems associated with each usability factor (eg, the number of issues related to the usability factor “design clarity” under the category “interface design.” To address our secondary objective, we noted which participants had observed specific problems, and counted how many were identified by both groups (those treated with immunotherapy and those not treated with immunotherapy) versus problems identified by only one group. Furthermore, for each distinct usability problem, we noted the number of participants who identified the problem and compared how frequently the problem was reported across the two participant groups.

We provide a more detailed account of the usability problems in the “Qualitative Assessment” section. We adopted a UCD approach and a postpositivist perspective. The eHealth Usability Problem Framework was used as a theoretical framework for our analysis. We included the problems most frequently identified, as well as those where there was a notable difference in identification between the two participant groups.

The qualitative analysis was conducted without the use of any specialized software. The quantitative analysis of counts and frequency was performed using Excel (Microsoft Corp).

### Perceived Usefulness and SUS Questionnaires

The SUS survey results were evaluated using the standard SUS scoring algorithm. We followed the general guideline for interpretation of the results, with scores below 68 indicating poor usability, and scores above 68 indicative of good usability. The Perceived Usefulness results were analyzed using frequency analysis.

## Results

### Participant Characteristics

We conducted 21 interviews in total. The interviews took place between June 2021 and April 2022. The average age of the participants was 53 (SD 12.1) years. We interviewed 7 patients with melanoma, 6 patients with renal cell carcinoma, 6 patients with other types of cancer (breast cancer, urinary cancer, lymphoma, and vestibular schwannoma), and 2 caregivers. See Table 3 for an overview of the participants’ characteristics. The participants with melanoma and renal cell carcinoma were treated with immune checkpoint inhibitors. All participants used a smartphone and were familiar with mobile apps. Five participants mentioned having used a health-related mobile app before.

**Table 3:** Characteristics of participants that were interviewed for this usability study (between June 2021 and April 2022).

			Treated with immunotherapy?		
			Yes (n=13)		No, N/A^a^ (caregiver; n=8)
**Sex, n (%)**					
	Male	6 (29)		6 (29)	
	Female	7 (33)		2 (10)	
**Age (years)**					
	Mean (SD)	52 (11)		56 (14)	
	Range	32-71		29-73	
**Type of cancer, n (%)**					
	Melanoma III-IV	7 (33)		0 (0)	
	Kidney I-II	4 (19)		0 (0)	
	Kidney III-IV	2 (10)		0 (0)	
	Breast I-II	0 (0)		1 (5)	
	Breast III-IV	0 (0)		2 (10)	
	Lymphoma	0 (0)		1 (5)	
	Urinary IV	0 (0)		1 (5)	
	Vestibular schwannoma I-II	0 (0)		1 (5)	
	N/A (caregiver)	0 (0)		2 (10)	
**Treatment status, n (%)**					
	On treatment	8 (38)		4 (19)	
	Off treatment	5 (24)		2 (10)	
	N/A (caregiver)	0 (0)		2 (10)	
**Living situation, n (%)**					
	Alone	2 (10)		1 (5)	
	With relatives	11 (52)		6 (29)	
	Shared housing	0 (0)		1 (5)	
**Type of smartphone, n (%)**					
	Android	6 (29)		5 (24)	
	iOS	7 (33)		3 (14)	

^a^Not applicable.

### Task Completion

We measured the effectiveness of the participants performing tasks by completion rates (Table 4). Participants found Task 1, “Go through introduction, check notifications, and report an activity,” and Task 3, “Report a fever symptom in a caregiver’s role,” the most challenging. Task 5, “Find and review information about skin toxicity,” had the highest completion rate. Participants who were not treated with immunotherapy seemed to struggle more with the symptom-reporting tasks (Tasks 2 and 3).

**Table 4:** Task completion rates of tasks performed during the think-aloud part of the usability interview, divided by participant group (participants previously treated with immunotherapy and not treated with immunotherapy).

	Task 1: introduction and home page		Task 2: symptom reporting and response inbox (itch as patient)			Task 3: symptom reporting and response inbox (fever as caregiver)			Task 4: coaching, goals, and activities			Task 5: patient education	
	Yes	No	Yes	No	Yes		No	Yes		No	Yes		No
Completed chemotherapy with ease, n (%)	8 (62)	5 (63)	11 (85)	4 (50)	10 (77)		3 (38)	9 (69)		5 (63)	11 (85)		8 (100)
Completed chemotherapy with difficulty, n (%)	5 (38)	3 (37)	2 (15)	4 (50)	3 (23)		5 (62)	4 (31)		3 (37)	2 (15)		0 (0)

### Usability Problems

In the four interviews used to assess data quality, no substantial differences were noted in the results from the Italian and Dutch research teams, nor were differences between the analyses based on transcripts compared to the analyses from notes. The participants identified 76 distinct usability problems and proposed 15 additional features missing in the CAPABLE app. The first task “Go through introduction, check notifications, and report an activity” resulted in 31 issues; the second and third tasks “Report an itch symptom in a patients’ role” and “Report a fever symptom in a caregivers’ role” resulted in 24 issues; the fourth task “Find and perform a breathing exercise” resulted in 16 issues; and the final fifth task “Find and review information about skin toxicity” resulted in 12 issues. Eight problems recurred in multiple tasks.

Table 5 shows an overview of the eHealth Usability Problem Framework [32] and the count of usability problems found per usability factor. The category with the highest number of usability problems is the task-technology fit category, which relates to the match between the system on the one hand, and the user, their context of use, and their health goals on the other hand. Of these, 23 usability problems were found by both participants who received immunotherapy and participants who did not receive immunotherapy, 31 problems were solely found by participants who received immunotherapy, and 22 problems were solely found by participants who did not receive immunotherapy. Table 6 shows an overview of all usability problems that were indicated by more than one participant, resulting in 32 problems. See Multimedia Appendix 4 for the complete table of usability problems. We discuss the usability problems that participants most frequently indicated during the study in the following section.

**Table 5:** Number of distinct usability problems identified during the think-aloud part of the usability interview^a^.

Usability factors		Number of issues found by both groups	Number of issues found only by the immunotherapy group	Number of issues found only by the nonimmunotherapy group	Number of total issues
**Basic system performance**		1	1	0	2
	General system interaction	1	1	0	2
**Task-technology fit**		6	9	7	22
	Fit between system and context of use	1	2	1	4
	Fit between system and health goals	3	2	3	8
	Fit between system and user	2	5	3	10
**Interface design**		2	5	4	11
	Design clarity	0	2	0	2
	Interface organization	1	1	1	3
	Readability of texts	1	0	2	3
	Symbols, icons, and buttons	0	2	1	3
**Navigation and structure**		4	0	1	5
	Navigation	3	0	1	4
	Structure	1	0	0	1
**Information and terminology**		6	3	6	15
	Health-related information	4	3	5	12
	System information	2	0	1	3
**Guidance and support**		1	7	1	9
	Procedural health-related information	1	6	1	8
	Procedural system information	0	1	0	1
**Satisfaction**		3	6	3	12
	Satisfaction with system	2	2	1	5
	Satisfaction with the system’s ability to support health goals	1	4	2	7
Total		23	31	22	76

^a^The counts are presented per usability factor and usability factor category.

**Table 6:** List of usability problems including the count of participants that identified these usability problems, divided by group (treated with immunotherapy vs not treated with immunotherapy)^a^.

			Treated with immunotherapy?	
			Yes	No
**Introduction and home page**				
	**Task-technology fit**			
		Current list of hobbies not sufficient	2	2
		Expects automatic detection of activities by smartwatch	3	1
		Time of going to bed is different every day	2	0
		Unclear what the added value is of recording exercises or activities	2	0
		Allow user to write down why they did or did not like the challenge	2	0
		Not possible to select multiple hobbies	1	1
		Need for balance between coaching, support for symptoms, and support for cancer treatment in content	0	2
	**Interface design**			
		Text is too long	5	2
		Participant prefers graphical explanations to textual explanations	0	2
	**Navigation and structure**			
		Link between times of waking up or going to bed and the symptom management unclear	1	1
	**Information and terminology**			
		Unclear what vital functions in home page are	2	0
	**Satisfaction**			
		Tone in the introduction text is not appreciated	1	1
**Symptom reporting and response inbox**				
	**Basic system performance**			
		Not clear that scrolling was necessary to view everything in screen	6	5
	**Task-technology fit**			
		Information missing about medication in recommendation (dosage, need for prescription, where to get it)	7	1
		Symptom descriptions do not match experience of itch of the patient, would be difficult to choose	6	0
		Feedback missing after report	1	5
	**Navigation and structure**			
		Participant cannot find symptom section easily	3	1
	**Information and terminology**			
		Term caregiver is unclear, caregiver can be a professional or family or friends visiting	1	1
		Guidance and support	0	0
		Unclear if the clinician will view the report, if patient will be contacted, and what is expected of patient	4	0
		Missing from instructions that caregivers can report symptoms	2	0
	**Satisfaction**			
		Symptom reporting process seems long or steps redundant	1	1
		Participant does not trust the feedback from the app	1	1
**Coaching, goals, and well-being interventions**				
	**Task-technology fit**			
		Expects automatic detection of activities by smartwatch	0	3
		Unclear how users can set their own goals	0	2
		Unmet expectation of the app recommending a schedule with activities, which can be personalized	2	2
	**Navigation and structure**			
		Participant cannot find the exercise easily	7	2
	**Information and terminology**			
		Names of types of breathing exercises are not self-explanatory	4	1
		Menu term “objectives or goals” does not match with content found	2	1
	**Guidance and support**			
		Unclear how goals relate to the activities	4	0
		Invitation for well-being intervention is not clear, not self-explanatory what it is and how to proceed	10	7
	**Satisfaction**			
		Content, purpose, and benefits of well-being interventions not sufficient currently	3	0
**Patient education**				
	**Interface design**			
		Reorganize the categories and structure of the educational content list	1	3
	**Navigation and structure**			
		Participant cannot find educational section easily (clicks on symptoms first)	1	3
		Information and terminology		
		First part of the text is difficult to understand without in-depth knowledge	1	2
		Education not the correct term for section	1	2
		Rash and itching are not translated	1	1
Total			128	85

^a^Problems included in this table were identified by at least two participants.

### Qualitative Assessment

#### Introduction and Home Page

Task-technology fit: the CAPABLE app asks the user to select their hobbies from a predefined list. This selection is used to recommend similar activities. Participants could not find their own hobbies in this list and were unable to select more than one hobby.

Interface design: in the introduction, the text to explain the purpose and functionalities of the CAPABLE app was deemed too long by some participants and participants stated a preference for more graphical explanations.

Yes, it [the text] was long, and the concentration of some patients might be decreased, so a video might be easier.

Participant 20

#### Symptom Reporting and Feedback Inbox

In general, it was not always clear to participants that scrolling was necessary to view everything on screen.

Task-technology fit: participants who received immunotherapy treatment predominantly experienced problems when reporting an “Itch” symptom using the symptom reporting function in the app. The app presents a set of symptom descriptions to choose from. Nearly half of the participants in the immunotherapy group found it difficult to choose one of those descriptions, which did not match their own experiences with itching caused by immunotherapy:

It’s very black-and-white, isn’t it? Yes, I had constant itching, all day long, but I could still do activities. And that’s not reflected in there. You really should give a lot more options ... I had constant itching, but of course, I could still go grocery shopping, and wash myself, and, sleeping was really difficult though, whole nights without sleep.

Participant 21

During the think-aloud task, the app recommended an emollient cream for the itch symptom reported by the participants. While participants appreciated the advice, a majority of the participants in the immunotherapy group found the message incomplete, as they were not sure what the exact dosage of the cream would be, if a prescription would be needed, and where to acquire the medication.

Guidance and support: after reporting the symptom, participants were confused about the next steps in the process. A subset of participants from the immunotherapy group expressed uncertainty on whether a clinician would view the report, in which cases they would be contacted by the clinician, and what was expected of them as a patient.

That may still need to be coordinated, like, when should the patient or caregiver take action themselves if they notice things are not going well, and when does the team proactively step in or call to check how things are going or if they can offer any help? would have expected if you report [symptoms] in this manner, that your care team would be informed.

Participant 15

#### Coaching, Goals, and Activities

Task-technology fit: the section “Goals” in the app contains a list of exercises and activities. These can be filtered by their goal, including supporting mental well-being, physical well-being, sleep, and acceptance. The connection between the name of the section and the list of activities was perceived as unclear and confusing by some participants. As a result, when asked to find a breathing exercise in the app, it was not always obvious for participants to click on the “Goals” button from the home page. In addition, while participants appreciated the ability to easily select and do an exercise, it was unclear how often to do these activities and how to schedule activities in the daily plan on the home page of the app:

[I expected] some sort of automatic coach within the system that informs you: you’ve chosen mental well-being as your focus, so every night before going to sleep, do fifteen minutes of meditation.

Participant 8

Participants were also confused about wearing a smartwatch while the app requires manual registration of exercises such as walking.

Guidance and support: users receive an invitation in the CAPABLE app inbox to participate in an activity, such as a daily walking challenge. While users are immediately prompted to accept this challenge, most participants found it unclear in the invitation what is expected of them, how they should proceed, where they can see the content of this program in the app, and whether and where they should register to do these activities.

#### Patient Education

Navigation and structure: participants were asked to find information about skin adverse events due to immunotherapy. Some participants had trouble finding the correct section in the app and clicked on the “Symptom management” section instead of the “Education” section.

Information and terminology: some participants indicated that the text in the example section shown during the interview was challenging to understand without in-depth knowledge.

### Perceived Usefulness and SUS Questionnaires

**Figure 3:**
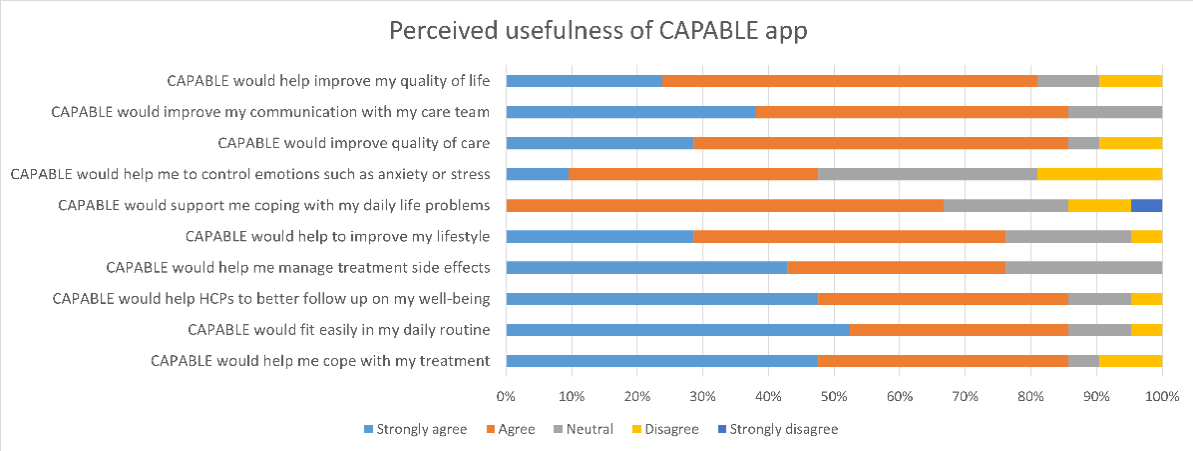
Outcomes of the Perceived Usefulness questionnaire filled in at the end of the usability interview by participants, presented as a stacked bar chart. CAPABLE: Cancer Patients Better Life Experience; HCP: health care provider.

The participants filled in a questionnaire aiming to measure their acceptance and perceived usefulness of the CAPABLE app. Overall, participants were positive about nearly all statements. For eight out of ten questions, more than 75% of participants agreed or strongly agreed (Figure 3). The statements that were most widely agreed upon were that the system would easily fit in users’ daily routines, could help health care professionals to follow up on patients’ well-being, could improve communication with the care team, could help users cope with their treatment, and could help users support their quality of life. Participants were more skeptical about the CAPABLE app helping to manage emotions such as anxiety and stress and the ability of the app to help cope with daily life problems. The results of the questionnaire were comparable between the participant group treated with immunotherapy, versus the group not treated with immunotherapy. Finally, the participants were asked to fill in the SUS questionnaire. The mean SUS score was 80 (SD 11), which is classified as “good” in terms of usability.

## Discussion

### Principal Findings

We evaluated the prototype of the CAPABLE app, a symptom monitoring and coaching system, with 21 participants divided into two groups, participants that had received immunotherapy (patients with melanoma and renal cell carcinoma) and that had not received immunotherapy (other cancer types), from The Netherlands and Italy. This evaluation was executed by use of think-aloud interviews and two questionnaires. In total, 76 usability issues were identified. Specifically, 23 usability issues were identified by both groups. The immunotherapy group found 31 additional issues, and the nonimmunotherapy group found 22 additional issues.

Most usability problems were in the task-technology fit category of the eHealth Usability Problem Framework [33]. This is reflected in problems encountered in the coaching section of the app. The participants’ mental model, defined as what a user knows, believes about, and expects from a system [34], did not match with the design and presentation of the digital behavior change interventions. Participants expected to be able to set their own goals, and thought that the app would suggest a recommended weekly schedule of activities that can be personalized according to their needs. Problems with task-technology fit were also encountered in creating a symptom report and with the subsequent advice given. Participants who had experienced itching due to immunotherapy found it difficult to choose one of the descriptions given to make a symptom report, as none of them matched their own experiences with the symptom. Additionally, participants felt that the advice provided was insufficient because it lacked information about the prescription, use, and dosage of the recommended medication, as well as whether to contact their clinician. This set of issues was mainly indicated by participants who had received immunotherapy. This is logical, since patients who received immunotherapy are more likely to have experienced a situation similar to the scenario used in the test, and could assess the fit of the app with their own lived experience. No differences were noted in the number and type of usability issues identified by caregivers compared to patients.

Despite identified usability issues, participants responded positively to the perceived acceptance and usefulness questionnaire regarding the CAPABLE app. Specifically, they would expect that the CAPABLE app would facilitate at-home monitoring, help patients cope with treatment, and support their quality of life.

Overall, our analysis of the usability problems seems to indicate a necessity to include participants with the characteristics of the intended end users for the evaluation of certain functionalities. In our case, this meant having the symptom-reporting functionality evaluated by patients who had been treated with immunotherapy, specifically with previous experience in immune-related adverse events. For the other tasks and functionalities, it seems likely that loosening the inclusion criteria would have resulted in sufficient feedback without critical missing usability issues.

### Interpretation and Impact

This study adds to previously published information by extracting a specific description of the usability problems encountered by intended end users during the evaluation of symptom management and coaching eHealth intervention. These findings are relevant for researchers developing and evaluating tools like symptom monitoring apps, especially for patients with cancer. More generally, we noted that patients who had taken immunotherapy noticed problems that other patients with cancer (and caregivers) did not. This is useful information for researchers who are considering whether patients who have experienced the specific health problem under study need to be recruited. It can be that these patients are quite ill, and researchers do not want to burden them. Our results suggest that the participation of these patients added valuable information, at least in this study, and thus asking for their participation in usability studies is justified.

### Comparison With Prior Work

This study found positive perspectives from participants on measured perceived acceptance and perceived usefulness. This might seem contradictory to the number and severity of usability issues reported. This is reflected in other studies, where participants are enthusiastic about the functionalities and perceived future benefits, while simultaneously encountering difficulties during the usability evaluation [35-37]. Patients’ wants and needs seem to be identified clearly in research, while a gap between user needs and eHealth tool implementation might be caused by practical considerations, the adaptability of the tool to local context, complexity factors, and health professionals’ uptake of the eHealth app [38].

In addition, we investigated the impact of different user characteristics on usability problems found during a think-aloud evaluation. Previous studies have researched the impact of domain knowledge, specified as the familiarity or expertise an individual has with a particular topic or subject area, on finding usability problems [39]. In these studies, the output of novices and experts, with previous expertise with the software or tools evaluated, was compared. In some cases, novices found more usability problems, less usability problems, or less usability problems but more severe problems, compared to experts [40-42]. However, this study would define previous domain knowledge not as previous experience with the software, but as previous experience with the treatment (immunotherapy) or previous experience with certain side effects (itch). Previous research found a significant difference between the novice and expert group with previous domain knowledge defined as previous pregnancy [43]. More research is needed on the impact of previous experience with a disease or treatment in the oncology field as part of the users’ profile on usability evaluation outcomes.

### Strengths and Limitations

In this study, we were able to recruit a large and diverse participant group from multiple centers in both The Netherlands and Italy. This was a benefit to our goal of collecting broad feedback on the CAPABLE tool and aiming to find a complete overview of potential usability issues. We had researchers from two different research teams performing the interviews. Nearly all researchers had previous interviewing experience, and a collaborative training session was held with all interviewers to streamline the UEMs used in the interviews as much as possible to prevent bias. The interviews were qualitative in nature; however, the data from the think-aloud tasks was specifically used to identify usability problems, rather than for thematic analysis. No pilot testing was performed, but no changes were needed during the course of the interview rounds.

The raw data used for coding the interviews varied: interviews with participants with renal cell carcinoma and other types of cancer were coded using summarizing interview notes, while interviews with melanoma participants were coded using transcriptions. This discrepancy may have led to missing data from incomplete interview notes. However, our data quality check revealed that although transcripts provided more context for the codes, the codes derived from the interview notes were consistent with those from the transcripts. In addition, we had multiple researchers involved in the coding and analysis phase of the study, to reduce the evaluator effect [44].

We did not take health, smartphone literacy, or physical or mental impairments into account during recruitment. There might be a selection bias of participants agreeing to the interview as they have an interest in using their mobile phones, or invitees refusing to participate because of limited smartphone literacy. However, we recruited a varied group of 21 participants, with varying ages, different diagnoses, past treatments, and countries.

### Future Work

The usability results of this study were used to improve the CAPABLE prototype. A prospective exploratory pilot study involving the final version of CAPABLE is currently being held in The Netherlands and Italy, with different patient populations treated with immune checkpoint inhibitors, including patients with melanoma and renal cell carcinoma [24]. The clinical impact and usability of the CAPABLE tool will be evaluated.

Our comparison of the usability problems generated by participants treated with immunotherapy versus the nonimmunotherapy group indicated a difference in output mainly for the symptom-reporting functionality of the CAPABLE tool. This comparison was based on qualitative data. Multiple factors may have been involved in the differences of usability problems found in addition to previous experience with the treatment or side effects, such as age, diagnosis, stadium of disease, health literacy, and country. However, our findings implore future studies to focus on exploring and comparing the usability evaluation output of participants with varying previous experiences. In addition, future research is needed to determine for which types of software, or which specific functionalities, there would be an added benefit or need for participants with specific characteristics.

### Conclusions

While participants identified usability problems regarding task-technology fit, interface design, and overall satisfaction, they responded positively regarding the perceived impact of CAPABLE in monitoring patients from home, helping to cope with treatment, and supporting quality of life. Further analysis of the usability problems indicates that it was essential to include participants who matched the target end users. Participants treated with immunotherapy, specifically with previous experience in immune-related adverse events encountered critical problems with symptom reporting that would not have been identified if these participants were not included. For other tasks and functionalities, it seems likely that loosening the inclusion criteria would have resulted in sufficient feedback without critical missing usability issues. Future research is needed to determine for which types of software and which specific functionalities, there would be an added benefit or need for participants with specific characteristics.

## Data Availability

The aggregated data is included in Multimedia Appendix 4. The unaggregated datasets generated during this study are not publicly available due to our institutions’ privacy policies but are available from the corresponding author upon reasonable request and with approval from the respective institution.

## Appendix

Multimedia Appendix 1 Consolidated criteria for reporting qualitative studies (COREQ): 32-item checklist.

Multimedia Appendix 2 Think-aloud interview protocol.

Multimedia Appendix 3 System Usability Scale and perceived impact questionnaire.

Multimedia Appendix 4 Overview of all usability problems and suggestions.

## Abbreviations

AIMAC: Italian Association of Cancer Patients, Relatives and Friends
CAPABLE: Cancer Patients Better Life Experience
COREQ: Consolidated Criteria for Reporting Qualitative Research
ICSM: Istituti Clinici Scientifici Maugeri
NKI-AvL: Netherlands Cancer Institute-Antoni van Leeuwenhoek
SUS: System Usability Scale
UCD: user-centered design
UEM: usability evaluation method
