# Supplementary material for: Usability and Usefulness of a Symptom Management Coaching System for Patients With Cancer Treated With Immune Checkpoint Inhibitors: Comparative Mixed Methods Study
Source: JMIR Form Res. 2025 Jan 23;9:e57659. doi: 10.2196/57659 (PMC11803325; doi:10.2196/57659)
Supplement: Multimedia Appendix 1 [file formative_v9i1e57659_app1.docx]

| **No. Item** | **Guide questions/description** | **Reported in section** |
| --- | --- | --- |
| **Domain 1: Research team and reﬂexivity** |  |  |
| *Personal Characteristics* |  |  |
| 1. Interviewer/facilitator | Which author/s conducted the inter view or focus group? | **Methods – Interviews** “The interviews were performed by two research teams. The interviews at NKI-AvL were performed in Dutch by two female PhD candidates with prior interviewing experience [I.F and S.G]. The interviews at ICSM and AIMAC were performed in Italian by a female researcher [V.T], a male senior researcher [M.O] and a male research assistant with previous experience as a healthcare professional [F.D].” |
| 2. Credentials | What were the researcher’s credentials? E.g. PhD, MD |  |
| 3. Occupation | What was their occupation at the time of the study? |  |
| 4. Gender | Was the researcher male or female? |  |
| 5. Experience and training | What experience or training did the researcher have? |  |
| *Relationship with participants* |  |  |
| 6. Relationship established | Was a relationship established prior to study commencement? | **Methods – Interviews** “Two participants from NKI-AvL had participated in a previous interview with the NKI-AvL research team to elicit specific needs and requirements for the CAPABLE tool. The researchers had no clinical relationship with nor did know the remaining participants”. |
| 7. Participant knowledge of the interviewer | What did the participants know about the researcher? e.g. personal goals, reasons for doing the research | **Methods – Interviews** “Participants were aware that the interviewers were researchers involved in the development of the CAPABLE prototype.” |
| 8. Interviewer characteristics | What characteristics were reported about the inter viewer/facilitator? e.g. Bias, assumptions, reasons and interests in the research topic |  |
| **Domain 2: study design** |  |  |
| *Theoretical framework* |  |  |
| 9. Methodological orientation and Theory | What methodological orientation was stated to underpin the study? e.g. grounded theory, discourse analysis, ethnography, phenomenology, content analysis | **Methods – Data analysis** “We adopted a user-centered design approach, and a post-positivist perspective. The eHealth Usability Problem Framework was used as a theoretical framework for our analysis.” |
| *Participant selection* |  |  |
| 10. Sampling | How were participants selected? e.g. purposive, convenience, consecutive, snowball | **See Table 1 in the Methods section.** For recruiting centers NKI and ICSM: “Purposive sampling strategy to obtain a sample that varied in age. Participants were invited by their treating clinician, face to face or by telephone”  For recruiting center AIMAC: “Open enrolment recruitment from volunteering patient network” |
| 11. Method of approach | How were participants approached? e.g. face-to-face, telephone, mail, email |  |
| 12. Sample size | How many participants were in the study? | **Results – Participant characteristics** “We conducted 21 interviews in total.” |
| 13. Non-participation | How many people refused to participate or dropped out? Reasons? | N/A |
| *Setting* |  |  |
| 14. Setting of data collection | Where was the data collected? e.g. home, clinic, workplace | **Methods – Interviews** “Due to the COVID-19 pandemic, interviews were done online using video-conferencing tools Microsoft TEAMS and Zoom.” |
| 15. Presence of non-participants | Was anyone else present besides the participants and researchers? | N/A |
| 16. Description of sample | What are the important characteristics of the sample? e.g. demographic data, date | **Methods – Recruiting and selection “**We aimed to recruit three participant groups; individuals diagnosed with melanoma, those with renal cell carcinoma, and participants without direct experience with the targeted cancer types (thus patients with other types of cancer or informal caregivers).” |
| *Data collection* |  |  |
| 17. Interview guide | Were questions, prompts, guides provided by the authors? Was it pilot tested? | **See Multimedia Appendix 1 for the interview protocol.**  **Discussion – Strengths and Limitations:** “No pilot testing was performed, but no changes were needed during the course of the interview rounds.” |
| 18. Repeat interviews | Were repeat inter views carried out? If yes, how many? | **Methods – Interviews** “Two participants from NKI-AvL had participated in a previous interview with the NKI-AvL research team to elicit specific needs and requirements for the CAPABLE tool. The researchers had no clinical relationship with nor did know the remaining participants”. |
| 19. Audio/visual recording | Did the research use audio or visual recording to collect the data? | **Methods – Interviews “**Due to the COVID-19 pandemic, interviews were done online using video-conferencing tools Microsoft TEAMS and Zoom. The interviews were recorded using the screen- and audio capture functionalities of these tools.” |
| 20. Field notes | Were ﬁeld notes made during and/or after the inter view or focus group? | **Methods – Data analysis** “The Dutch interview recordings at NKI-AvL were transcribed verbatim [S.G] as source data for our coding and analysis of the usability problems. During the Italian interviews at ICSM and AIMAC, notes were made by the interviewer that summarized the participants’ feedback. These notes were revised and completed after the interview upon reviewing the recording.” |
| 21. Duration | What was the duration of the inter views or focus group? | **Methods – Interviews “**The planned duration of the interviews was 45-60 minutes**.”** |
| 22. Data saturation | Was data saturation discussed? | **Methods – Recruiting and inclusion** “We did not specifically aim for data saturation as we considered the recommended sample sizes found in the literature, which range from five to ten participants.” |
| 23. Transcripts returned | Were transcripts returned to participants for comment and/or correction? | **Methods – Data analysis** “The transcripts and notes were not returned to participants.” |
| **Domain 3: analysis and ﬁndings** |  |  |
| *Data analysis* |  |  |
| 24. Number of data coders | How many data coders coded the data? | **See Figure 1 for an overview of the steps executed for the coding of interview recordings**. **Two researchers were involved in coding and labeling the data. Data was coded with the aim to extract usability problems. The usability problems were mapped to an existing framework.** |
| 25. Description of the coding tree | Did authors provide a description of the coding tree? |  |
| 26. Derivation of themes | Were themes identiﬁed in advance or derived from the data? |  |
| 27. Software | What software, if applicable, was used to manage the data? | **Methods – Data analysis** “The qualitative analysis was conducted without the use of any specialized software.” |
| 28. Participant checking | Did participants provide feedback on the ﬁndings? | **No.** |
| *Reporting* |  |  |
| 29. Quotations presented | Were participant quotations presented to illustrate the themes/ﬁndings? Was each quotation identiﬁed? e.g. participant number | **See Results – Qualitative assessment** |
| 30. Data and ﬁndings consistent | Was there consistency between the data presented and the ﬁndings? | **See Discussion – Principle findings** |
| 31. Clarity of major themes | Were major themes clearly presented in the ﬁndings? | N/A |
| 32. Clarity of minor themes | Is there a description of diverse cases or discussion of minor themes? | N/A |
