# Supplementary material for: Usability and Usefulness of a Symptom Management Coaching System for Patients With Cancer Treated With Immune Checkpoint Inhibitors: Comparative Mixed Methods Study
Source: JMIR Form Res. 2025 Jan 23;9:e57659. doi: 10.2196/57659 (PMC11803325; doi:10.2196/57659)
Supplement: Multimedia Appendix 2 [file formative_v9i1e57659_app2.docx]

# Introduction of the interview

## Introducing the study

We are very pleased that you wish to participate in this interview and study. You should have received an information letter about the study, do you have any questions about the letter?

## Purpose of the study

With this study, we would like to find out what users of the CAPABLE app for patients and the CAPABLE dashboard for doctors think of the functionalities and the user-friendliness of CAPABLE. Your contribution to this study is important, as your opinions about the app will help us develop and improve the app to meet the needs of (multiple) patients. In the future we hope to have a well-developed app, which can help patients during their treatment in an easy and user-friendly manner.

## Topics

Based on previous research on health apps, there are a number of topics we will discuss with you. We will start with general questions about you and your lifestyle habits. Next, we will give a general explanation of the app and specific explanations of the functionalities of the app. We will then ask you to open the prototype on your computer. We will ask you to perform a number of tasks using this prototype and explain what you think and experience while performing these tasks. We will ask you to fill in a questionnaire about the impact of the CAPABLE app. Then, we will ask you to fill in a questionnaire about the user-friendliness of the CAPABLE app. Finally, we will ask you if you miss any information and conclude the interview.

## Confidentiality

To protect your privacy, we give your data a code. On all your data, we will only put this code. We keep the key to the code in a secure place at [**insert hospital]**. When we process your data, we always use only this code. Apart from the research staff and the people mentioned in the next paragraph, nobody will be able to see your encrypted data. Also, in reports and publications about the research, nobody can find out any information about you. We will keep your data and video recordings for 20 years or as long as scientifically relevant, after which we will delete them. We will keep them so that we can look at the recording again at a later date (e.g. after receiving additional information from other interviews) to see if we have missed any things. All data and video recordings will be stored on a secure disk at the **[insert hospital]**.

## Stopping the interview

If during the interview you decide you do not want to continue, you can say so at any time. We will then stop the interview. The information we have collected up to that point will be used for the study.

## Recording

We will use recording software from our computer to record the interview. The law states that we must ask you for your permission before using recording software. I will therefore ask you in a moment, when the recording is on, if you give permission for this conversation to be recorded. Do you consent to this interview being recorded?

## Stating tasks of the interviewers

I will conduct the interview.

# Patient profile

- What is your relationship with new technologies?
- Do you have a smartphone? *Yes/no*
  - What kind of smartphone do you have?
  - For which purposes?
  - How often?
- Do you use the internet? *Yes/no*
  - For which purposes?
  - How often?

# Introduction to CAPABLE

## General explanation of CAPABLE

The CAPABLE app is a coaching and information system for patients and healthcare professionals, developed for use during and after immunotherapy treatment. The main goal of the support provided by CAPABLE is to improve the quality of life of patients. CAPABLE consists of a mobile app for patients and a dashboard on the computer for doctors. We will now briefly explain the goals and functionalities of CAPABLE and ask your opinion about each goal.

## Goal 1: Providing information

A first goal of the CAPABLE app is to help patients during and after immunotherapy treatment, by providing information about the disease, treatment and side-effects. We hope this will give patients a better understanding of the decisions and treatments that affect their disease and health. The app will therefore cover topics such as diagnosis, treatments, side-effects, etc.

- What do you think about this goal?
- Would you like to use this app functionality?
- Do you have anything else to add?

## Goal 2: Support in mental and physical health

A second goal of the CAPABLE app is to help patients with maintaining a healthy lifestyle and to support patients with their mental and physical well-being during treatment. The app will contain different topics, developed in collaboration with oncologists but also nutritionists, psychologists and experts in the field of healthy food and physical activity habits, etc. For topics such as exercise, nutrition, sleep, mindfulness and so on, the app will suggest both information and activities you can follow. These topics are sometimes related, such as how physical activity and mindfulness could possibly improve sleep, and how the right nutrition can help with dealing with certain side-effects, and so on.

- What do you think about this goal?
- Would you like to use this app functionality?
- Do you have anything else to add?

## Goal 3: Symptom monitoring

Furthermore, a third goal of the CAPABLE app is to ease the communication between patient and doctor about wellbeing, symptoms, side-effects and vital functions. CAPABLE will do this by giving patients the possibility to fill out questionnaires, enter symptoms in the app themselves, and wear a sensor like a FitBit to communicate vital signs. All information filled in by the patient in his or her CAPABLE app, subsequently arrives at the CAPABLE dashboard of the doctor, who can act upon it and contact the patient if necessary. The CAPABLE app itself can also give patients advice and information about the symptoms and how to deal with them.

- What do you think about this goal?
- Would you like to use this app functionality?
- Do you have anything else to add?

## Trying out the CAPABLE app

We have developed a first version of the CAPABLE app, and would like to ask you to try it out. We are going to ask you to carry out three tasks in the app, one by one ,and while doing so to tell us aloud what comes to mind. These are the tasks: Enter two new symptoms. View new notifications in the app. Go to your recommended activities for today and complete them. The app is still under development. In a short while, we will send you a link that will take you to the test version of the app. You can open the link via your computer. You can click on the screen of the app. If you press certain buttons, the app will show a new page, just as apps normally do on a smartphone. The buttons and parts of the screen you can click on within this app prototype, are colored blue.

| **Task 1 Introduction, notifications and activity** | |
| --- | --- |
| Duration (seconds) |  |
| Description of scenario | Go through the introduction, check the notifications and report the activity suggested in the notifications. |
| Think-aloud comments |  |
| How easy was the task?  *1-5 very difficult – very easy* |  |
| Do you have any remarks? |  |

| **Task 2 Itch symptom** | |
| --- | --- |
| Duration (seconds) |  |
| Description of scenario | Report an itch symptom in the patient role |
| Think-aloud comments |  |
| How easy was the task?  *1-5 very difficult – very easy* |  |
| Do you have any remarks? |  |

| **Task 3 Fever symptom** | |
| --- | --- |
| Duration (seconds) |  |
| Description of scenario | Report a fever symptom in the caregiver role |
| Think-aloud comments |  |
| How easy was the task?  *1-5 very difficult – very easy* |  |
| Do you have any remarks? |  |

| **Task 4 Exercise** | |
| --- | --- |
| Duration (seconds) |  |
| Description of scenario | Find and perform a deep breathing exercise |
| Think-aloud comments |  |
| How easy was the task?  *1-5 very difficult – very easy* |  |
| Do you have any remarks? |  |

| **Task 5 Information** | |
| --- | --- |
| Duration (seconds) |  |
| Description of scenario | Find and review information about skin toxicity |
| Think-aloud comments |  |
| How easy was the task?  *1-5 very difficult – very easy* |  |
| Do you have any remarks? |  |

# Follow-up questions

- Do you think that there are missing functionalities in the CAPABLE app?
- Do you have any suggestions to improve the CAPABLE app?
