# Supplementary material for: Usability and Usefulness of a Symptom Management Coaching System for Patients With Cancer Treated With Immune Checkpoint Inhibitors: Comparative Mixed Methods Study
Source: JMIR Form Res. 2025 Jan 23;9:e57659. doi: 10.2196/57659 (PMC11803325; doi:10.2196/57659)
Supplement: Multimedia Appendix 3 [file formative_v9i1e57659_app3.docx]

| **System Usability Scale questionnaire** | | | | | |
| --- | --- | --- | --- | --- | --- |
| I will now ask you ten questions about the usability of the CAPABLE app. On a scale of 1-5, please note the extent to which you agree or disagree with each of the following statements (1 = Strongly Agree and 5 = Strongly Disagree) | | | | | |
|  | **Strongly agree** | **Agree** | **Neutral** | **Disagree** | **Strongly disagree** |
| I think that I would like to use this system frequently. |  |  |  |  |  |
| I found the system unnecessarily complex. |  |  |  |  |  |
| I thought the system was easy to use. |  |  |  |  |  |
| I think that I would need the support of a technical person to be able to use this system. |  |  |  |  |  |
| I found the various functions in this system were well integrated. |  |  |  |  |  |
| I thought there was too much inconsistency in this system. |  |  |  |  |  |
| I would imagine that most people would learn to use this system very quickly. |  |  |  |  |  |
| I found the system very cumbersome to use. |  |  |  |  |  |
| I felt very confident using the system. |  |  |  |  |  |
| I needed to learn a lot of things before I could get going with this system. |  |  |  |  |  |

| **Perceived impact questionnaire** | | | | | |
| --- | --- | --- | --- | --- | --- |
| I will ask you ten questions about the usefulness and impact of the CAPABLE app. Could you tell us the extent to which you agree or disagree with each of the following statements, from a scale from 1 to 5, from strongly agree to strongly disagree (1 = Strongly agree and 5 = Strongly disagree) | | | | | |
|  | **Strongly agree** | **Agree** | **Neutral** | **Disagree** | **Strongly disagree** |
| CAPABLE would help me cope with cancer treatment |  |  |  |  |  |
| CAPABLE would easily fit in my everyday life |  |  |  |  |  |
| CAPABLE would help doctors to better monitor patients during treatment |  |  |  |  |  |
| CAPABLE would help me to manage side effects of the treatment |  |  |  |  |  |
| CAPABLE would help me improve my lifestyle |  |  |  |  |  |
| CAPABLE would support me to cope with daily life problems |  |  |  |  |  |
| CAPABLE would help me to control my emotions (anxiety, stress) |  |  |  |  |  |
| CAPABLE would improve the quality of health care |  |  |  |  |  |
| CAPABLE would improve communication of patients with their health professional team |  |  |  |  |  |
| CAPABLE would help improve my quality of life |  |  |  |  |  |
