# Supplementary material for: Usability and Usefulness of a Symptom Management Coaching System for Patients With Cancer Treated With Immune Checkpoint Inhibitors: Comparative Mixed Methods Study
Source: JMIR Form Res. 2025 Jan 23;9:e57659. doi: 10.2196/57659 (PMC11803325; doi:10.2196/57659)
Supplement: Multimedia Appendix 4 [file formative_v9i1e57659_app4.docx]

Multimedia Appendix 4 Overview of all usability problems and suggestions

Table 1 Overview of all usability problems and suggestions. Number of distinct usability problems indicated per participant group (immunotherapy yes/no).

|  | **Treated with immunotherapy?** | |  |
| --- | --- | --- | --- |
|  | **Yes** | **No** | **Total** |
| **Basic system performance** | **6** | **4** | **10** |
| **general system interaction** | **6** | **4** | **10** |
| not clear that scrolling was necessary to view everything in screen | 5 | 4 | 9 |
| unclear how to end the exercise | 1 |  | 1 |
| **Task-technology fit** | **27** | **18** | **45** |
| **fit between system and context of use** | **5** | **3** | **8** |
| expects automatic detection of activities by smartwatch | 3 | 2 | 5 |
| need for balance between coaching, support for symptoms, and support for cancer treatment in content |  | 1 | 1 |
| possibility to connect smartphone sensors to app | 1 |  | 1 |
| include caregiver's name and phonenumber for the clinician | 1 |  | 1 |
| **fit between system and health goals** | **10** | **10** | **20** |
| information missing about medication in recommendation (dosage, need for prescription, where to get it) | 5 | 1 | 6 |
| feedback missing after report | 1 | 4 | 5 |
| unmet expectation of the app recommending a schedule with activities, with options for personalisation by users | 2 | 2 | 4 |
| unclear how users can set their own goals |  | 1 | 1 |
| unclear what the added value is of recording capsules | 1 |  | 1 |
| activities are recommended based on the users' current interests, new suggestions are missing | 1 |  | 1 |
| emotional status is not included in symptom report |  | 1 | 1 |
| app seems like a personal trainer, misses link with oncologists |  | 1 | 1 |
| **fit between system and user** | **12** | **5** | **17** |
| symptom descriptions do not match experience of itch of the patient, would be difficult to choose | 3 |  | 3 |
| current list of hobbies not sufficient | 2 | 1 | 3 |
| time of going to bed is different every day | 2 |  | 2 |
| not possible to select multiple hobbies | 1 | 1 | 2 |
| allow user to write down why they did or did not like the challenge | 2 |  | 2 |
| user tried to select more than one symptom |  | 1 | 1 |
| feedback provided by the smartwatch (e.g. reminders) should be configurable by the user |  | 1 | 1 |
| difficult to choose time of going to bed (between the time ranges) | 1 |  | 1 |
| notes that list is not yet complete, fatigue symptom is missing | 1 |  | 1 |
| misses information about treatments other than immunotherapy |  | 1 | 1 |
| **Interface design** | **11** | **9** | **20** |
| **design clarity** | **2** |  | **2** |
| include illustrations and icons | 1 |  | 1 |
| does not notice the inbox notification | 1 |  | 1 |
| **interface organisation** | **2** | **2** | **4** |
| reorganize the categories and structure of the educational content list | 1 | 1 | 2 |
| uncertainty about showing too many activities in daily plan | 1 |  | 1 |
| filters in the inbox are too distracting |  | 1 | 1 |
| **readability of texts** | **5** | **6** | **11** |
| text is too long | 5 | 2 | 7 |
| participant prefers graphical explanations to textual explanations |  | 3 | 3 |
| repitition in the text |  | 1 | 1 |
| **symbols, icons, and buttons** | **2** | **1** | **3** |
| expects information to drop-down when clicking on the header | 1 |  | 1 |
| participant had to be instructed to add the free-text note |  | 1 | 1 |
| graphic should represent sex of patient | 1 |  | 1 |
| **Navigation and structure** | **10** | **8** | **18** |
| **navigation** | **9** | **7** | **16** |
| participant cannot find capsule easily | 5 | 2 | 7 |
| participant cannot find symptom section easily | 3 | 1 | 4 |
| participant cannot find educational section easily (clicks on symptoms first) | 1 | 3 | 4 |
| unclear how to get back to main screen |  | 1 | 1 |
| **structure** | **1** | **1** | **2** |
| link between times of waking up/going to bed and the symptom management unclear | 1 | 1 | 2 |
| **Information and terminology** | **12** | **13** | **25** |
| **health-related information** | **9** | **10** | **19** |
| names of types of breathing exercises are not self-explanatory | 2 | 1 | 3 |
| first part of the text is difficult to understand without in-depth knowledge | 1 | 2 | 3 |
| unclear what vital functions in homepage are | 2 |  | 2 |
| term caregiver is unclear, caregiver can be a professional or family/friends visiting | 1 | 1 | 2 |
| rash and itching are not translated | 1 | 1 | 2 |
| term unclear, replace gravita with intensita |  | 1 | 1 |
| unclear who is part of the careteam | 1 |  | 1 |
| the referenced link is in english. include a translated version |  | 1 | 1 |
| highlight in the introduction that the doctor can see the data between one visit to another |  | 1 | 1 |
| term eruzione cutanea unclear |  | 1 | 1 |
| acronyms are confusing |  | 1 | 1 |
| replace "emolliente topico da banco" with easier synonym | 1 |  | 1 |
| **system information** | **3** | **3** | **6** |
| menu term 'objectives/goals' does not match with content found | 2 | 1 | 3 |
| education' not the correct term for section | 1 | 1 | 2 |
| privacy management missing |  | 1 | 1 |
| **Guidance and support** | **21** | **5** | **26** |
| **procedural health-related information** | **20** | **5** | **25** |
| CAPSULE invitation is not clear, not self-explanatory what the CAPSULE is and how to proceed | 9 | 4 | 13 |
| should be clear if a clinician will view the symptom report, if the patient will be contacted, and what the patient should do | 3 |  | 3 |
| unclear how goals relate to the activities | 3 |  | 3 |
| include in instructions that a caregiver can report a symptom and check off that they reported the symptom. | 2 |  | 2 |
| unclear how the participant will be reminded of the walking activity | 1 |  | 1 |
| feedback missing on how to find an activity that is suitable for user if they indicate to not like the activity | 1 |  | 1 |
| include pop-up after symptom report that message will arrive in inbox | 1 |  | 1 |
| include an introduction to the section |  | 1 | 1 |
| **procedural system information** | **1** |  | **1** |
| participant would like to flag/report more than one symptom at a time | 1 |  | 1 |
| **Satisfaction** | **11** | **6** | **17** |
| **satisfaction with system** | **4** | **3** | **7** |
| symptom reporting process seems long/steps redundant | 1 | 1 | 2 |
| tone in the introduction text is not appreciated | 1 | 1 | 2 |
| frequency of feedback - at the end of the challenge (after 30 days) or perhaps every week, not daily | 1 |  | 1 |
| user interface currently bare-bones | 1 |  | 1 |
| too much administrative effort to use the app |  | 1 | 1 |
| **satisfaction with system's ability to support health goals** | **7** | **3** | **10** |
| content, purpose and benefits, of CAPSULES not sufficient currently | 3 |  | 3 |
| participant does not trust the feedback from the app | 1 | 1 | 2 |
| risk that proposed activities are not compatible with preferences of the users. | 1 |  | 1 |
| misses general content in education section |  | 1 | 1 |
| unclear how to "complete" exercise for sleeping if the purpose is falling asleep | 1 |  | 1 |
| content of educational section should be expanded | 1 |  | 1 |
| pursuing goals (mental and physical wellbeing, nutrition) would require in-person support |  | 1 | 1 |
| **Additional outcomes** | **12** | **7** | **19** |
| **proposed functionality** | **12** | **7** | **19** |
| include additional videos and animations about treatment and for the capsules | 2 | 1 | 3 |
| include push notifications for activities | 2 |  | 2 |
| possibility of syncing the capable calendar with a calendar | 1 | 1 | 2 |
| add link to existing sources | 1 |  | 1 |
| add link to aimac forum |  | 1 | 1 |
| add guiding voice audio to meditation capsules | 1 |  | 1 |
| add link to guidelines |  | 1 | 1 |
| include search function for symptom list | 1 |  | 1 |
| misses information about fake news |  | 1 | 1 |
| add information about meditation | 1 |  | 1 |
| recommends to add a stepcounter in the app |  | 1 | 1 |
| sending voice-messages can be interesting, specially from the clinical team. |  | 1 | 1 |
| would appreciate possibility to send messages to healthcare professional | 1 |  | 1 |
| include functionality to exchange opinions with peers with the same pathology | 1 |  | 1 |
| include hospital appointments in daily plan | 1 |  | 1 |
| **Grand Total** | **110** | **70** | **180** |
